# A French multicenter randomised trial comparing two dose-regimens of prothrombin complex concentrates in urgent anticoagulation reversal

**DOI:** 10.1186/cc11923

**Published:** 2013-01-10

**Authors:** Delphine Kerebel, Luc-Marie Joly, Didier Honnart, Jeannot Schmidt, Damien Galanaud, Claude Negrier, Friedrich Kursten, Pierre Coriat, Lex206 Investigator Group

**Affiliations:** 1 French Army Hospital Sainte-Anne, 2 Boulevard Saint-Anne, 83000 Toulon, France; 2 CHU Rouen, 1 Rue de Germont, 76000 Rouen, France; 3 CHU Dijon, 4 Rue Gaffarel, 21079 Dijon, France; 4 CHU Clermont-Ferrand, 58 Rue Montalembert, 63000 Clermont-Ferrand, France; 5 CHU Pitié-Salpêtrière, 47-83 Boulevard de l'Hôpital, 75651 Paris, France; 6 CHU Lyon, 103 Grande Rue de la Croix-Rousse, 69004 Lyon, France; 7 Octapharma AG, Oberlaaer Strasse 235, 1100 Vienna, Austria

## Abstract

**Introduction:**

Prothrombin complex concentrates (PCC) are haemostatic blood preparations indicated for urgent anticoagulation reversal, though the optimal dose for effective reversal is still under debate. The latest generation of PCCs include four coagulation factors, the so-called 4-factor PCC. The aim of this study was to compare the efficacy and safety of two doses, 25 and 40 IU/kg, of 4-factor PCC in vitamin K antagonist (VKA) associated intracranial haemorrhage.

**Methods:**

We performed a phase III, prospective, randomised, open-label study including patients with objectively diagnosed VKA-associated intracranial haemorrhage between November 2008 and April 2011 in 22 centres in France. Patients were randomised to receive 25 or 40 IU/kg of 4-factor PCC. The primary endpoint was the international normalised ratio (INR) 10 minutes after the end of 4-factor PCC infusion. Secondary endpoints were changes in coagulation factors, global clinical outcomes and incidence of adverse events (AEs).

**Results:**

A total of 59 patients were randomised: 29 in the 25 IU/kg and 30 in the 40 IU/kg group. Baseline demographics and clinical characteristics were comparable between the groups. The mean INR was significantly reduced to 1.2 - and ≤1.5 in all patients of both groups - 10 minutes after 4-factor PCC infusion. The INR in the 40 IU/kg group was significantly lower than in the 25 IU/kg group 10 minutes (*P *= 0.001), 1 hour (*P *= 0.001) and 3 hours (*P *= 0.02) after infusion. The 40 IU/kg dose was also effective in replacing coagulation factors such as PT (*P *= 0.038), FII (*P *= 0.001), FX (*P *<0.001), protein C (*P *= 0.002) and protein S (0.043), 10 minutes after infusion. However, no differences were found in haematoma volume or global clinical outcomes between the groups. Incidence of death and thrombotic events was similar between the groups.

**Conclusions:**

Rapid infusion of both doses of 4-factor PCC achieved an INR of 1.5 or less in all patients with a lower INR observed in the 40 IU/kg group. No safety concerns were raised by the 40 IU/kg dose. Further trials are needed to evaluate the impact of the high dose of 4-factor PCC on functional outcomes and mortality.

**Trial registration:**

Eudra CT number 2007-000602-73.

## Introduction

Strong evidence supports the use of oral anticoagulant therapy (OAT) in primary and secondary prophylaxis of venous thromboembolism and in patients with prothrombotic factors such as atrial fibrillation and prosthetic heart valves [1,2]. An estimated 1 to 1.5% of the western population has undergone OAT, including mainly vitamin K antagonists (VKA), following the publication of guidelines in favour of anticoagulation [3,4].

However, VKAs carry a significant risk of life-threatening haemorrhage including intracerebral and subdural haemorrhage. Thirty-day acute mortality for VKA-associated intracranial haemorrhage is high, ranging from 40 to 60% [5]. Most survivors are neurologically impaired and suffer from severe disability [6,7]. Haematoma volume and lower level of consciousness are major determinants of a poor prognosis in these patients [7,8] and up to 50% of patients develop secondary volume enlargement [9].

Timely medical management of VKA-associated intracranial haemorrhage, including immediate discontinuation and rapid reversal of the anticoagulant therapy, is crucial to improve patient prognosis by reducing haematoma growth [10]. OAT reversal is achieved by replacing depleted coagulation factors by using fresh frozen plasma (FFP) or prothrombin complex concentrate (PCC) and by administering vitamin K. The goal of these measures is to decrease the international normalised ratio (INR) values to ≤1.4 and preferably ≤1.2 [10-17].

Four of the current guidelines recommend PCC rather than FFP as the first-choice treatment to increase levels of vitamin K-dependent coagulation factors [10-18]. A review of the published literature found that PCCs, compared to FFP, were more effective in shortening the time to INR correction and were associated with a low risk of thrombotic adverse events [19,20].

A recent meta-analysis including 1,032 patients supports the safety of PCC in patients with VKA-associated intracranial haemorrhage and reports an incidence of thromboembolic events of 1.4% (95% CI, 0.8 to 2.1) [21]. The safety and efficacy of PCC were also demonstrated by the rapid infusion speed in these patients [22-24].

The early PCC composition contained three coagulation factors II, IX and X, with no or very little amount of factor VII. However, newer formulations, available in some European countries, include normal amounts of factor VII and are thus known as 'four-factor concentrates' or '4-factor PCCs'. The optimal dose of PCC for OAT reversal has not yet been established. Doses ranging between 25 and 50 international units (IU)/kg have been recommended, since 1 IU/kg raises factors II, IX and × levels by about 1% and a 30 to 50% increase in factors levels are usually considered sufficient to achieve haemostasis [13].

Recent results suggest that higher doses could provide clinical benefit in terms of INR normalization and subsequently in term of clinical outcomes, without increasing the risk of thromboembolic complications [23]. The aim of this randomised study was to compare the efficacy and safety of two dose regimens of 4-factor PCC, 25 and 40 IU/Kg, in emergency reversal of anticoagulation in patients with intracranial haemorrhage.

## Materials and methods

### Overall design and study plan

This phase III, multicentre, randomised, open-label study included two parallel groups and was conducted in 22 centres in France. Of these, 16 centres enrolled at least one patient. This study was conducted in compliance with the Helsinki Declaration. The protocol was submitted for approval to an ethics committee and to the French drug regulatory body (AFSSAPS- French Agency for Health Product Safety). Ethics and regulatory approvals were obtained before the start of inclusions. The necessary written informed consent was obtained for all patients involved in the study, including consent to publish.

### Study population

Patients were eligible for inclusion if they had an objectively diagnosed OAT-associated cerebral haemorrhage (computed tomography or magnetic resonance). Other inclusion criteria were age ≥18 years and written informed consent. When a patient was unable to sign the informed consent, consent was obtained from a legal representative or a family member. Exclusion criteria were deep coma on admission (Glasgow Coma Scale (GCS) 3 to 5), generalised seizure, acute sepsis, crush injury or disseminated intravascular coagulation, high risk of thrombosis (pulmonary embolism or phlebitis during the last three months), concomitant disability (modified Rankin scale (mRS) >2), patients having received vitamin K prior to admission to the investigational centre, known allergy to vitamin K, hypersensitivity to the active substances of 4-factor PCC (human coagulation factors II, VII, IX and X) or to any of its excipients (heparin and sodium citrate), known allergy to heparin or history of heparin-induced thrombocytopenia, participation in another clinical study currently or during the past three months, pregnancy or breast feeding.

### Study treatment

Included patients received 25 or 40 IU/kg body weight of 4-factor PCC (Octaplex™, Octapharma AG, Lachen, Switzerland). The study treatment is a human plasma-derived concentrate that contains vitamin K-dependant clotting factors II, VII, IX and × as well as protein C and S. The product also contains heparin and citrate added during the manufacturing process. The treatment was administered immediately in an emergency setting after randomisation and usually before any INR results were available. A centralised randomisation was done via a dedicated website. The randomisation was stratified according to the type of cerebral haemorrhage (intracerebral or subdural). For each type of cerebral haemorrhage, a randomly permuted block scheme was used, with a variable and undisclosed block size. All patients were treated concomitantly with an intravenous infusion of 5 mg vitamin K. A PCC rescue dose was administered if the targeted INR (≤1.5) was not reached, 10 min after the end of the infusion. An additional infusion of 4-factor PCC was allowed at intervals of 6 h after the administration of the first dose, if the INR remained >2.

### Study outcomes

The primary endpoint of the study was the mean INR at 10 min after the end of 4-factor PCC infusion in each group. Secondary endpoints included changes in INR, prothrombin time (PT), coagulation factors, protein C and protein S, haematoma growth, clinical status (GCS), overall clinical response 48 h after the end of infusion and global clinical outcome assessed using extended Glasgow Outcome Scale (GOS-E), modified Rankin Scale (mRS) and Barthel Index (BI) at day 30 after infusion. GOS-E is a global scale for functional outcome that rates patient status into one of five categories: dead, vegetative state, severe disability, moderate disability or good recovery. The mRS is a score for measuring the degree of disability or dependence in the daily activities, ranging from 0 (no symptoms) to 6 (dead). BI is a scale used to measure performance in basic activities of daily living, ranging from 0 (totally dependent) to 100 (independent).

Safety endpoints were the occurrence of adverse events (AE), particularly thromboembolic events, and changes in vital signs, ECG and laboratory parameters. The occurrence of any AE - including death, thromboembolic complications, intracranial haematoma recurrence and allergic reactions- as well as the need for neurosurgery, were monitored throughout hospital stay and for 30 days after the infusion.

### Laboratory and clinical assessments

At inclusion, all patients underwent a complete clinical assessment that included medical history, physical examination and determination of vital signs. Blood samples were collected to measure INR, PT, coagulation factors II, VII, IX and X, protein C and protein S prior to infusion, and at 10 ± 5 min, 1, 3, 6 and 24 h after the end of the infusion.

Medical imaging was done 48 h after the end of the infusion or earlier in case of neurological worsening. Clinical status was evaluated at 1, 24 and 48 h.

### Sample size and statistical analysis

All statistical analyses were performed using SAS software (SAS Institute Inc., Cary, NC, USA). Descriptive statistics were performed by dose group (25 and 40 IU/kg) for all parameters and were expressed as percentages or averages with standard deviation (SD). A descriptive post hoc analysis was performed by infusion speed group (<8 ml/min and ≥8 ml/min). All efficacy analyses were performed in intention to treat. Groups were compared using Student's *t *test or Wilcoxon rank sum test for quantitative efficacy criteria, and Pearson's χ^2 ^or Fisher's exact test for qualitative efficacy criteria. Safety analyses were presented by dose group. All statistical tests were two-sided and *P *<0.05 was considered statistically significant. For sample size calculation, the following considerations were performed. The minimal difference expected between the two groups INR at 10 ± 5 min was 0.4, with a standard deviation of 0.30. Considering an alpha error of 0.05 with a power of 0.95, required sample size for a one-sided *t *test was 13 patients in each group. However, a larger population size of 30 patients in each group was included for the comparison of baseline and post-treatment values between groups.

## Results

### Baseline characteristics of study population

Between November 2008 and April 2011, 59 patients were included and randomised: 29 in the 4-factor PCC 25 IU/kg group and 30 in the 40 IU/kg group. Almost all patients (n = 53) were admitted in emergency units, three were admitted in intensive care units, one in a neurology unit and one in a neurosurgery unit. Premature withdrawal occurred in fourteen patients, six in the 25 IU/kg group and eight in the 40 IU/kg group. Main reasons were death (n = 8), patient transfer to another facility (n = 3), lost to follow-up (n = 2) and discharge from hospital (n = 1). However, these patients were present for the primary criteria evaluation (INR at 10 min after the end of the infusion). All the patients were included in the results analysis.

Baseline characteristics of the patients are reported in Table 1. Overall mean age was 76.4 ± 10.3 years and 71.2% were male patient. Patient characteristics did not differ between the 25 and 40 IU/kg groups. All patients had a cardiovascular history. History of neurological and endocrine disorders was present in 62.7% and 52.5% of patients, respectively. All patients were treated with vitamin K antagonist: 85% were under oral anticoagulant with fluindione, 12% with warfarin sodium and 3% with acenocoumarol. No marked difference was found between dose groups.

**Table 1:** Demographic and clinical characteristics at inclusion.

	4-factor PCC regimen		
		
	25 IU/kg (n = 29)	40 IU/kg (n = 30)	*P *value
**Age, years**	77.7 ± 9.4	75.2 ± 11.1	0.265
**Male gender**	19 (65.5)	23 (76.7)	0.344
**Body weight, kg**	76.9 ± 17.6	76.7 ± 13.5	0.779
**Medical history**			NS
Hypertension	18 (62.1)	21 (70.0)	
Atrial fibrillation	18 (62.1)	18 (60.0)	
Previous ischemic stroke	3 (10.3)	4 (13.3)	
Diabetes mellitus	8 (27.6)	7 (23.3)	
Hypercholesterolemia	3 (10.3)	5 (16.7)	
**Blood pressure, mm Hg**			
SBP	154 ± 23.4	162 ± 32.7	0.359
DBP	84.1 ± 14.0	86.2 ± 18.3	0.524
**Blood glucose, mmol/L**	7.7 ± 3.1	7.6 ± 2.4	0.946
**Onset to diagnosis, hrs**	29.4 ± 51.6	45.8 ± 82.0	0.213
**INR**	2.9 ± 1.1	3.2 ± 1.9	0.660
**GCS**	13.3 ± 2.7	13.2 ± 2.8	0.846
**Type of intracranial haemorrhage**			0.862
Intracerebral haemorrhage	18 (64.3)	18 (62.1)	
Acute subdural haemorrhage	10 (35.7)	11 (37.9)	
**Haematoma volume, cm^3^**	23.8 ± 18.5	16.5 ± 18.9	0.438

Results are expressed as n (%) or mean ± SD. NS, not significant. GCS, Glasgow Coma Scale; INR, international normalised ratio; PCC, prothrombin complex concentrate.

Median INR at inclusion was 2.8 (range 1.3 to 11.4) and median GCS was 15 (range 6.0 to 15.0). Mean time from symptoms onset to diagnosis was 37.6 ± 68.4 hours. Intracranial haemorrhage at inclusion was intracerebral in 36 patients (63.2%) and subdural in 21 patients (36.8%). Mean haematoma volume was evaluated in 19 patients and was 20.3 ± 18.5 cm3. Haematoma volume was comparable between dose groups.

### Treatment infusion

All patients received the allocated dose of 4-factor PCC at randomisation. The total IU of infused PCC ranged from 1270 to 4000. Treatment was administered over a mean infusion time of 10 min (SD 6, range 3 to 30).

The mean administered dose was 1837 IU per patient (SD 433, range 1270 to 2500) in the 25 IU/kg group and 3017 IU per patient (SD 448, range 2500 to 4000) in the 40 IU/kg group. Mean duration of infusion was significantly higher in the 40 IU/kg group compared with the 25 IU/kg group (12.3 ± 7.0 vs. 8.4 ± 4.7 min, *P *= 0.021). No rescue dose was administrated. However, only one patient, belonging to the 40 IU/kg group, received one additional infusion at 6 h, as the INR exceeded 2 at this time point.

A total of sixteen patients with subdural haemorrhage underwent neurosurgical intervention for evacuation of the haematoma: eight patients in the 25 IU/kg group and eight in the 40 IU/kg group. There were no significant differences between dose groups for neurosurgery characteristics.

### Response to treatment

The overall median INR at inclusion was 2.8 (range 1.30 to 11.4). INR was similar in both dose groups at inclusion: 2.5 (range 1.3 to 6.0) for the 25 IU/kg group and 2.8 (range 1.7 to 11.4) for the 40 IU/kg group (*P *= 0.660). At 10 min after 4-factor PCC infusion, median INR was significantly reduced to 1.2 (range 1.0 to 1.5), declining to ≤1.5 in all patients in both groups. A target INR, defined as INR ≤1.2, was achieved in 44.5% of the 25 IU/kg group and in 76.0% of the 40 IU/kg group. The overall median INR values at inclusion, 10 min, 1, 3, 6 and 24 h were 2.8, 1.2, 1.2, 1.2, 1.2 and 1.1, respectively. The benefit of treatment infusion was maintained over time until at least 24 h.

**Figure 1:**
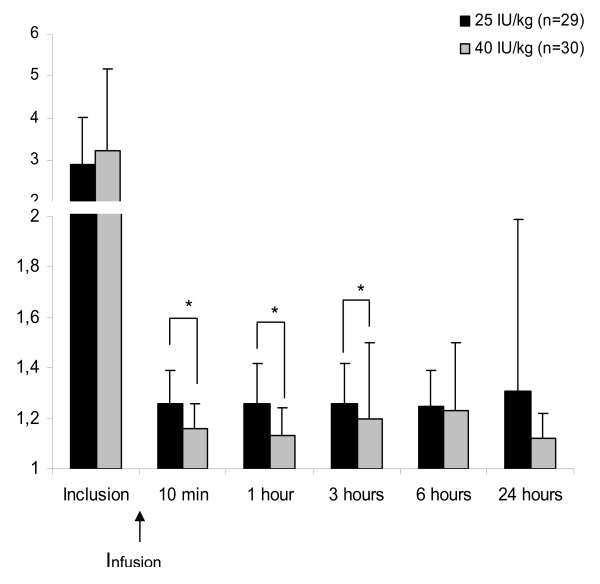
**Changes in mean INR values after 4-factor PCC infusion in 25 and 40 IU/kg groups**. *Significant difference with *P *<0.05. All patients were present during the 24 h. At inclusion, INR was calculated for 29 patients in the 25 IU/kg group and 30 patients in the 40 IU/kg group, at 10 min in 27 and 29, at 1 h in 26 and 28, at 3 h in 28 and 30, and at 24 h in 22 and 30, respectively. INR, international normalised ratio; PCC, prothrombin complex concentrate.

The change over time is shown in Figure 1 in the 25 and 40 IU/kg groups. A significant difference was found between dose groups: INR in the 40 IU/kg group was significantly lower than in the 25 IU/kg group at 10 min (*P *= 0.001), 1 h (*P *= 0.001) and 3 h (*P *= 0.02) after infusion. These results showed a dose-dependent effect of 4-factor PCC dose on INR at 10 min.

At 10 min after 4-factor PCC infusion, the INR in intracerebral haemorrhage and acute subdural haemorrhage subgroups was also dependent on the dose. Mean INR was significantly lower with the 40 IU/kg dose in intracerebral haemorrhage (1.14 ± 0.09 vs.1.23 ± 0.12 in the 25 IU/kg group, *P *= 0.015) and in acute subdural haemorrhage (1.17 ± 0.11 vs. 1.33 ± 0.11 in the 25 IU/kg group, *P *= 0.007).

**Figure 2:**
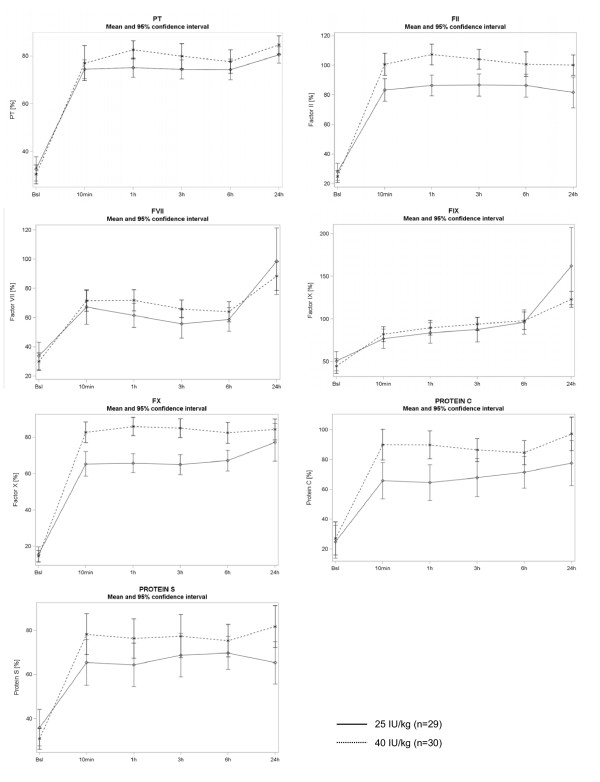
**Changes in coagulation factors after 4-factor PCC infusion in 25 and 40 IU/kg groups**. *Results are presented as mean and 95% confidence interval. PCC, prothrombin complex concentrate.

The impact of 4-factor PCC infusion was also demonstrated on PT and coagulation factors FII, FVII, FIX, FX, protein C and protein S. At 10 min after infusion, median PT value increased from 31.6% at inclusion to 77.0%, FII from 24.0% to 89.0%, FVII from 27.7% to 65.4%, FIX from 44.1% to 79.8%, FX from 12.4% to 74.0%, protein C from 15.5% to 81.2% and protein S from 32.0% to 71.0%. This established therapeutic effect was maintained over time until at least 24 h. Evolution of coagulation factors is presented in Figure 2 in the 25 and 40 IU/kg groups.

Significant differences were found between dose groups for PT (*P *= 0.038), FII (*P *= 0.001), FX (*P *<0.001), protein C (*P *= 0.002) and protein S (0.043) at 10 min after infusion. At 10 min after infusion, these values were higher and closer to normal measures in the 40 IU/kg group compared with the 25 IU/kg group. A dose of 40 IU/kg tended to be more effective in normalising PT and coagulation factors than a 25 IU/kg dose.

### Overall clinical outcome

At 48 h, mean haematoma volume was 31.1 ± 31.9 cm^3 ^in the 19 evaluated patients. There was no significant difference in haematoma volume between dose groups (33.2 ± 33.9 in the 25 IU/kg vs. 28.7 ± 31.4 in the 40 IU/kg, *P *= 0.713).

All clinical outcomes at 48 h and day 30 after infusion are presented in Table 2 for both dose groups.

**Table 2:** Overall clinical outcome at 48 hours and day 30 after infusion, in 25 and 40 IU/kg groups.

	4-factor PCC regimen		
		
	25 IU/kg (n = 29)	40 IU/kg (n = 30)	*P *value
**Overall clinical response - verbal rating scale at 48 h**			1.000
None	2 (8.0)	2 (7.1)	
Moderate	6 (24.0)	7 (25.0)	
Excellent	17 (68.0)	19 (67.9)	
**GCS**			
At 48 hours	13.1 ± 3.8	13.1 ± 3.8	0.724
At day 30	12.8 ± 4.0	13.3 ± 3.2	0.858
GOS-E at day 30*			0.460
Dead	2 (7.7)	4 (14.3)	
Vegetative state	2 (7.7)	1 (3.6)	
Lower severe disability	10 (38.5)	11 (39.3)	
Upper severe disability	2 (7.7)	4 (14.3)	
Lower moderate disability	1 (3.8)	1 (3.6)	
Lower good recovery	2 (7.7)	5 (17.9)	
Upper good recovery	7 (26.9)	2 (7.1)	
**mRS at day 30**	3.0 ± 2.0	3.4 ± 2.0	0.342
**BI at day 30**	53.4 ± 40.2	46.0 ± 43.2	0.623

Results are expressed as n (%) or mean ± SD. *GOS-E was performed for a total of 54 patients: 26 patients in the 25 IU/kg group and 28 patients in the 40 IU/kg group, which explains the difference in number of deaths with that reported in the text (n = 10). BI, Barthel Index; GCS, Glasgow Coma Scale; mRS, modified Rankin Scale; GOS-E, extended Glasgow Outcome Scale; PCC, prothrombin complex concentrate.

Mean Glasgow Coma Scale (GCS) was 13.1 ± 3.76 at 48 h and 13.1 ± 3.60 at day 30 after infusion. No difference in GCS was found between dose groups at 48 h (*P *= 0.724) and day 30 (*P *= 0.858). The overall clinical response was assessed as excellent in the majority of patients (67.9% with 95% confidence interval (CI) (55.4 to 8.5)) and moderate in 24.5% (95% CI (12.9 to 36.1)). Overall clinical response was similar between dose groups.

At day 30 after infusion, nine patients (16.7%) were dead or in a vegetative state, twenty-seven (50.0%) with severe disability, two (3.7%) with moderate disability and sixteen (29.7%) with good recovery, according to the GOS-E assessment. Overall, mean mRS was 3.20 ± 2.01 and mean BI was 49.7 ± 41.5 at day 30, meaning that patients were moderately disabled. No difference in mRS or in BI was found between dose groups.

### Safety

All adverse events (AE) and serious adverse events (SAE) are presented in Table 3 for the 25 and 40 IU/kg groups. Within 30 days of follow-up after infusion, ten patients died: four in the 25 IU/kg group and six in the 40 IU/kg group. No difference was found in mortality between dose groups (*P *= 0.731). The causes of death were as follows: haematoma evolution (n = 3), cerebral haemorrhage (n = 3), increased intracranial pressure (n = 1), brain stem ischemia (n = 1), pneumonia (n = 1) and hyperthermia (n = 1). Two deaths were considered as unlikely to be related to 4-factor PCC infusion and the other deaths were considered as unrelated to 4-factor PCC infusion. Of the two deaths considered unlikely to be related to PCC infusion, the first patient died 7 days after the infusion from a major persistent hyperthermia with neurovegetative disorders; and the second died 3 days after the infusion subsequent to a progression of the haematoma.

**Table 3:** Safety and incidence of AE and SAE in 25 and 40 IU/kg groups.

	4-factor PCC regimen		
		
	25 IU/kg (n = 29)	40 IU/kg (n = 30)	*P *value
**Death**	4 (13.8)	6 (20.0)	0.731
**Patients with at least one AE**	24 (82.8)	25 (83.3)	1.000
**Patients with at least one SAE**	11 (37.9)	12 (40.0)	0.871
**Patients with at least one TE**	2 (6.9)	2 (6.7)	1.000

Results are expressed as n (%). AE, adverse effects; PCC, prothrombin complex concentrate; SAE, serious adverse effects; TE, thrombotic event.

Four thrombotic events (TE) occurred during the study: two ischemic strokes in the 25 IU/kg group, and pulmonary embolism and phlebitis in the 40 IU/kg group. The two ischemic strokes occurred 3 and 18 days after the PCC infusion. Pulmonary embolism and phlebitis occurred 7 and 9 days after the PCC infusion, respectively. The medical history of these patients included mainly atrial fibrillation, hypertension, hypercholesterolemia, obesity, and past thromboembolic event. These TEs were judged, by the investigators, to be unrelated to 4-factor PCC infusion.

A further eighteen SAE occurred in fifteen patients: eight in the 25 IU/kg group and ten in the 40 IU/kg group. One SAE was considered as unlikely to be related to the study infusion and the others were considered as not related to the study infusion. The incidence of AE and SAE was similar between dose groups.

### Post hoc analysis: results by infusion speed

**Figure 3:**
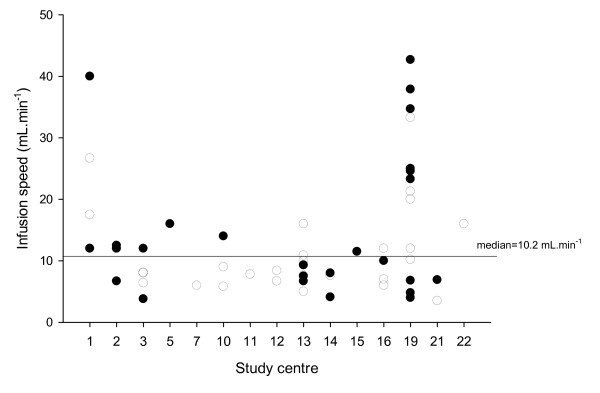
**Individual patient infusion speed at each study centre**. White dots refer to patients in the 25 IU/kg group and black dots refer to those in the 40 IU/kg group.

Infusion speed was between 3 and 8 mL min^-1 ^in 20 patients and ≥8 mL min^-1 ^in 39 patients (range: 3.5 to 42.7 mL min^-1^). The median infusion speed was 10.2 mL min^−1 ^(interquartile range (IQR), 6.9 to 16.0 mL min^−1^), equivalent to 254.0 IU min^−1 ^(IQR, 171.9 to 400.0 IU min^−1^). The respective minimum and maximum individual patient values were 3.5 and 42.7 mL min^−1^. Figure 3 shows the individual patient infusion speeds, which were between 3 and 8 mL min^−1 ^in 20 patients (34%) and ≥8 mL min^−1 ^in 39 (66%). Infusion speed exceeded 30 mL min^−1 ^in five patients, of whom four were enrolled at one study centre.

At baseline, mean INR was 3.35 ± 2.16 in the lower infusion speed group and 2.93 ± 1.19 in the higher infusion speed group. At 10 ± 5 min after infusion, the mean INR was significantly reduced to ≤1.5 in both groups. The occurrence of AEs was 90% (18/20) in patients with infusion speed between 3 and 8 ml/min vs. 80% (31/39) in patients with infusion speed ≥8 ml/min.

## Discussion

This randomised study demonstrates that the 40 IU/kg dose of 4-factor PCC is more effective than the 25 IU/kg dose in producing rapid reduction in INR as well as in replacing coagulation factors. Furthermore, the safety of the 40 IU/kg dose was found to be similar to that of the 25 IU/kg dose: no significant AEs were reported in either group during the immediate post-infusion period and the longer term safety was supported by a low death rate and few thrombotic events reported at the three-month follow-up. Of note, this study showed lower mortality rate compared with those reported in previously published papers that approached 60% in patients with OAT-associated intracranial haemorrhage [20]. Regardless of the intracranial haemorrhage type, subdural or intracerebral haemorrhage, and despite the rapid infusion speed (maximum infusion speed was 42.7 ml/min); 4-factor PCC infusion was effective and safe.

The content of 4-factor PCC (Octaplex™) used in this study is well balanced [25]. It contains the vitamin K-dependent clotting factors II, VII, IX, and × in proportions similar to those occurring in plasma. The other vitamin K-dependent proteins of the clotting cascade, protein S and C, are present in therapeutically effective concentrations to provide antithrombotic effects. Finally, it contains also small amounts of heparin to prevent activation of clotting factors.

When PCCs are administered without vitamin K, usually a rapid normalization of the INR with a rebound increase is observed 12 to 24 h later; this rebound increase in the INR is routinely seen when coagulation factor replacement is not accompanied by vitamin K [26]. The reason is that the half-life of vitamin K far exceeds the half-life of the administered PCC. Up to date, there are no clear recommendations concerning the dose of vitamin K in the literature (between 5 and 10 mg in French guidelines). Actually, our results showed that 5 mg of vitamin K is sufficient to maintain normal coagulation after 6 h in both groups. This role of vitamin K explains the absence of the statistical differences at 6 and 24 h between groups.

There is an ongoing debate about the therapeutic targeted INR in the setting of life-threatening intracranial haemorrhage. Some guidelines recommend that a target INR below 1.5 should be achieved, while others advise a target INR between 1 and 1.2. This is probably of interest for intracranial haemorrhage. All the patients in our study had achieved an INR ≤1.5 within 10 min after infusion. However, 76% of the patients in the 40 IU/kg group reached an INR ≤1.2 as opposed to only 44.5% in the 25 IU/kg group. The individual levels of clotting factors as well as protein C and S showed a good and long-lasting recovery for both dose groups. However, better recovery was obtained with 40 IU/kg 4-factor PCC for PT, factors II and X, and protein C. No significant difference in factor VII and factor IX was found between the groups. The large extravascular distribution of factor IX could explain this result. At 24 h, the observed increase in factors VII and IX could be due to the relay of vitamin K endogenous synthesis. The relationship between INR and clotting factors remains unclear. A recent *in vitro *study showed a correlation between INR correction and factors replacement after 4-factor PCC addition [27]. Achieving an INR below 1.5 does not always seem to be correlated with appropriate factors replacement. Optimising the therapeutic regimen towards a more individualized dosing based on weight is likely to correct INR more accurately and to elevate factors to appropriately safe levels [27,28].

Baseline large haematoma volume (>30 ml) and haematoma growth are predictive of poorer outcome [29]. Data on haematoma growth in patients treated for VKA-associated intracranial haemorrhage are limited. A recent report indicated that immediate INR reversal with 4-factor PCC is required to prevent haematoma growth [30]. In our study, no differences in clinical outcomes including haematoma volume and clinical indicators were observed between the groups, although the higher dose was more effective on INR normalization. This may be due to the small sample size and to the study design: although our data showed a haematoma volume growth, the results are limited by the small proportion of patients (n = 19) who underwent imaging at 48 h. Imaging was not imposed by the protocol and it was performed only in case of neurologic worsening.

According to the French guidelines, the PCC infusion was administered in an emergency before any INR results were available. Results showed that this management can prevent wasting time waiting for the initial INR value and guarantees a complete reversal of anticoagulation within 10 min, allowing consequently early surgery if necessary, as for example in case of subdural haematoma.

Generally, management of VKA-associated intracranial haemorrhage depends on the patient's symptoms severity. In this study, patients who died within 30 days had received the infusion in a shorter time lapse after admission than those who survived (1.25 vs. 2.50 h, *P *= 0.007). This was primarily related to the severity of haemorrhage at admission: patients who died were more severe at admission (GSC = 10 vs. 14 for survivals, *P *= 0.001). These data reflect the real-life practical management of VKA-associated intracranial haemorrhage in emergency care. Patients are managed according to their prognostic factors.

In France, the standard dose indicated for the 4-factor PCC is 25 IU/kg. A single-centre trial conducted in 18 patients with VKA-associated intracranial haemorrhage showed that 20 IU/kg of 4-factor PCC was effective in reversing the effects of anticoagulation immediately [24]. Its safety was also demonstrated since no haemorrhagic or thrombotic adverse effect was observed [24]. Another study including 60 patients confirmed the efficacy and safety of the 4-factor PCC at a median total dose of 41.1 (15.3 to 83.3) IU/kg [23].

There are a few limitations that need to be acknowledged and addressed regarding the present study. First, patient inclusion was not consecutive because of the study was conducted in an emergency setting and because of the availability of the product. Second, a surrogate laboratory primary endpoint was used instead of a clinical endpoint (for example, death). Other limitations include small sample size and open-label design.

## Conclusions

To our knowledge, this is the largest randomised study that has specifically evaluated the efficacy and safety of a 40 IU/kg dose of 4-factor PCC, compared with a 25 IU/kg in the management of VKA-associated intracranial haemorrhage. The dose of 40 IU/kg was more effective in reducing INR to a value ≤1.2. No difference in TE was noticed between the groups. However, around 25% of patients who received the high dose of 40 IU/kg did not reach the target INR (≤1.2). In a retrospective study, an INR ≥1.3 was significantly associated with a higher risk of 30-day mortality in the subgroup of patients with intracranial haemorrhage [31]. Although this study design was debatable, this result suggests that normalisation of the INR (to value <1.2) could improve the survival [30]. On the other hand, other authors advocate for an INR <1.5, meaning that this issue remains undefined [31]. While the impact of the 40 IU/kg dose on clinical outcomes was not conclusive in our study, our results support the need for further larger trials to be conducted to evaluate the impact of high-dose 4-factor PCC on functional outcomes and mortality.

## Key messages

• The 40 IU/kg dose of 4-factor PCC was more effective in reducing the INR to above 1.2 than the 25 IU/kg dose.

• A long-lasting benefit on clotting factor elevation was also observed with the 40 IU/kg dose for PT, factors II and X, and protein C.

• No difference was found in the haematoma volume at 48 h after infusion, or in the global clinical outcomes. This might be related to the study design.

• Safety of the 40 IU/kg dose of 4-factor PCC was demonstrated. The incidence of thrombotic events or serious adverse events was similar between groups.

## Abbreviations

AE: adverse event; BI: Barthel Index; CI: confidence interval; FFP: fresh frozen plasma; GCS: Glasgow Coma Scale: GOS-E: extended Glasgow Outcome Scale; INR: international normalised ratio; IQR: interquartile range; IU: international unit; mRS: modified Rankin Scale; OAT: oral anticoagulant therapy; PCC: prothrombin complex concentrate; PT: prothrombin time; SAE: serious adverse event; SD: standard deviation; TE: thrombotic event; VKA: vitamin K antagonist.

## Competing interests

Delphine Kerebel, Luc-Marie Joly, Didier Honnart, Jeannot Schmidt, Damien Galanaud, Claude Negrier and Pierre Coriat received honorarium from Octapharma, for their participation in the study. Pr Pierre Coriat was the study coordinator. Delphine Kerebel, Luc-Marie Joly, Didier Honnart and Jeannot Schmidt were the major investigators. Damien Galanaud performed the centralised scanners review and Claude Negrier performed the centralised laboratory services. Friedrich Kursten is an employee of Octapharma.

## Authors' contributions

DK, LMJ, DH, JS were the majors investigators and drafted the manuscript; DG participated in the design of the study, performed the centralized scanners review and helped to draft the manuscript. CN participated in the design of the study, performed the centralised laboratory services and helped to draft the manuscript. FK participated in the design of the study, performed the statistical analysis and the study management and helped to draft the manuscript. PC conceived of the study, and participated in the design and coordination, and helped to draft the manuscript. All authors read and approved the final manuscript.
